# Supplementary material for: Silibinin and SARS-CoV-2: Dual Targeting of Host Cytokine Storm and Virus Replication Machinery for Clinical Management of COVID-19 Patients
Source: J Clin Med. 2020 Jun 7;9(6):1770. doi: 10.3390/jcm9061770 (PMC7356916; doi:10.3390/jcm9061770)
Supplement: Supplementary file 1 [file jcm-09-01770-s001.pdf]

SUPPLEMENTARY INFORMATION

Table S1. Details of the interaction of silibinin docked to SARS-CoV-2 nsp12 (6NUR-based homology model)

Cluster number	ΔG, [kcal/mol]	Dissoc. constant, [μM]	Members	Residues of nsp12 (model) that contact silibinin
1	-9.641	0.08571	12%	ARG 553, ARG 555, TRP 617, ASP 618, TYR 619, CYS 622, ASP 623, ARG 624, THR 680, SER 682, THR 687, ASN 691, ASP 760, ASP 761, ALA 762, GLU 811, SER 814, ASP 920, THR 921, SER 922, ASN 923, ASP 924
2	-9.327	0.14562	4%	ASP 452, ASN 497, LYS 500, SER 501, LYS 545, ARG 553, ALA 554, ARG 555, THR 556, VAL 557, ALA 558, GLY 559, VAL 560, ASP 623, ARG 624, SER 682, GLY 683, ASP 920, SER 922
3	-9.185	0.18506	3%	ARG 553, ASP 618, TYR 619, CYS 622, ASP 623, THR 680, SER 682, THR 687, ASN 691, ASP 760, ASP 761, LYS 798, TRP 800, GLU 811, PHE 812, CYS 813, SER 814, ASP 920, THR 921, SER 922, ASN 923, ASP 924
4	-8.83	0.33692	1%	LYS 500, LYS 545, ARG 569, GLN 573, LEU 576, LYS 577, ALA 580, ASP 623, THR 680, SER 681, SER 682, ASP 684, ALA 685, THR 687, ALA 688, TYR 689, ASN 691, SER 759, ASP 760, ASP 920, THR 921, SER 922, ASN 923, ASP 924

For the best-docked silibinin molecule of each cluster, the Gibbs free energy, the dissociation constant, the number of molecule members (as %), and the residues that contact silibinin are shown. Catalytic residues are marked in red whereas those involved in the mechanism of action of nucleotide analogs are in green.

Table S2. Details of the interaction of remdesivir docked to SARS-CoV-2 nsp12 (6NUR-based homology model)

Cluster number	ΔG, [kcal/mol]	Dissoc. constant, [μM]	Members	Residues of nsp12 (model) that contact remdesivir
1	-9.38	0.13316	17%	ASP 452, TYR 455, LYS 545, ARG 553, ALA 554, ARG 555, THR 556, VAL 557, LYS 621, CYS 622, ASP 623, ARG 624, THR 680, SER 682, THR 687, ASN 691, SER 759, ASP 760, ASP 920, THR 921, SER 922, ASN 923, ASP 924
2	-8.906	0.29636	2%	ASP 452, LYS 500, LYS 545, ARG 553, ALA 554, ARG 555, THR 556, VAL 557, LYS 621, CYS 622, ASP 623, ARG 624, THR 680, SER 682, ASP 684, ALA 685, THR 687, ALA 688, TYR 689, ASN 691, SER 759, ASP 760, ASP 920, THR 921, SER 922, ASN 923, ASP 924
3	-8.814	0.34614	1%	ARG 553, ARG 555, GLY 616, TRP 617, ASP 618, TYR 619, LYS 621, CYS 622, ASP 623, THR 680, SER 682, THR 687, ASN 691, SER 759, ASP 760, ASP 761, ALA 762, TRP 800, GLU 811, PHE 812, CYS 813, SER 814, ASP 920, THR 921, SER 922, ASN 923, ASP 924

For the best-docked remdesivir molecule of each cluster, the Gibbs free energy, the dissociation constant, the number of molecule members (as %), and the residues that contact silibinin are shown. Catalytic residues are marked in red whereas those involved in the mechanism of action of nucleotide analogs are in green.

Table S3. Details of the interaction of ribavirin docked to SARS-CoV-2 nsp12 (6NUR-based homology model)

Cluster number	ΔG, [kcal/mol]	Dissoc. constant, [µM]	Members	Residues of the nsp12 (model) that contact ribavirin
1	-7.358	4.04124	18%	ASP 452, TYR 456, MET 542, ARG 553, ALA 554, ARG 555, THR 556, VAL 557, ALA 558, ASP 623, ARG 624, LYS 676, THR 680, SER 681, SER 682, ASP 920, THR 921, SER 922

For the best-docked ribavirin molecule of each cluster, the Gibbs free energy, the dissociation constant, the number of molecule members (as %), and the residues that contact silibinin are shown. Catalytic residues are marked in red whereas those involved in the mechanism of action of nucleotide analogs are in green.

Table S4. Details of the interaction of sofosbuvir docked to SARS-CoV-2 nsp12 (6NUR-based homology model)

Cluster number	ΔG, [kcal/mol]	Dissoc. constant, [µM]	Members	Residues of the nsp12 (model) that contact sofosbuvir
1	-8.523	0.56566	20%	ASP 452, TYR 456, MET 542, LYS 545, ARG 553, ALA 554, ARG 555, THR 556, VAL 557, ALA 558, CYS 622, ASP 623, ARG 624, THR 680, SER 681, SER 682, THR 687, ASN 691, SER 759, ASP 760, ASP 920, THR 921, SER 922, ASN 923, ASP 924

For the best-docked sofosbuvir molecule of each cluster, the Gibbs free energy, the dissociation constant, the number of molecule members (as %), and the residues that contact silibinin are shown. Catalytic residues are marked in red whereas those involved in the mechanism of action of nucleotide analogs are in green.

Table S5. Details of the interaction of silibinin docked to SARS-CoV-2 nsp12 (6M71)

Cluster number	DG, [kcal/mol]	Dissoc. constant, [µM]	Members	Residues of the nsp12 (6M71) that contact silibinin
1	-8.424	0.66854	12%	ASP 164, VAL 166, GLU 167, LYS 551, GLY 616, TRP 617, ASP 618, TYR 619, PRO 620, LYS 621, ASP 761, VAL 792, PHE 793, MET 794, SER 795, LYS 798, CYS 799, TRP 800, GLU 811, PHE 812, CYS 813, SER 814
2	-8.342	0.76777	6%	GLY 616, TRP 617, ASP 618, TYR 619, PRO 620, LYS 621, CYS 622, ASP 623, ASP 760, ASP 761, ALA 797, LYS 798, CYS 799, TRP 800, HIS 810, GLU 811, PHE 812
3	-7.819	1.85608	2%	ILE 494, ASN 496, LYS 500, ARG 569, GLN 573, LEU 576, LYS 577, ALA 580, ASP 623, THR 680, SER 681, SER 682, ASP 684, ALA 685, THR 687, ALA 688, TYR 689, ASN 691, SER 759

For the best-docked silibinin molecule of each cluster, the Gibbs free energy, the dissociation constant, the number of molecule members (as %), and the residues that contact silibinin are shown. Catalytic residues are marked in red whereas those involved in the mechanism of action of nucleotide analogs are in green.

Table S6. Details of the interaction of silibinin docked to SARS-CoV-2 nsp12 (7BTF)

Cluster number	ΔG, [kcal/mol]	Dissoc. constant, [µM]	Members	Residues of the nsp12 (7BTF) that contact silibinin
1	-8.21	0.95938	1%	PRO 169 A LEU 172 A ARG 249 A VAL 315 A SER 318 A THR 319 A ARG 349 A GLU 350 A THR 394 A CYS 395 A PHE 396 A ARG 457 A ASN 459 A LEU 460 A PRO 461 A THR 462 A ASN 628 A MET 629 A VAL 675 A LYS 676 A PRO 677
2	-8.147	1.06701	15%	ARG 553, ARG 555, THR 556, GLY 616, TRP 617, ASP 618, TYR 619, PRO 620, LYS 621, CYS 622, ASP 623, ARG 624, ASN 691, ASP 760, ASP 761, LYS 798, TRP 800, HIS 810, GLU 811
3	-7.837	1.80054	2%	ASP 452, TYR 455, ARG 553, THR 556, TRP 617, ASP 618, TYR 619, PRO 620, LYS 621, CYS 622, ASP 623, ARG 624, ASP 760, ASP 761, LYS 798, CYS 799, TRP 800, GLU 811
4	-7.8	1.91657	1%	ILE 494, VAL 495, ASN 496, LYS 500, LYS 545, ARG 555, VAL 557, ARG 569, GLN 573, LEU 576, LYS 577, ALA 580, SER 682, GLY 683, ASP 684, ALA 685, THR 687, ALA 688, TYR 689
5	-7.797	1.92630	1%	LYS 545, ARG 553, ARG 555, THR 556, VAL 557, TYR 619, PRO 620, LYS 621, CYS 622, ASP 623, ARG 624, THR 680, SER 681, SER 682, THR 687, ALA 688, ASN 691, SER 759

For the best-docked silibinin molecule of each cluster, the Gibbs free energy, the dissociation constant, the number of molecule members (as %), and the residues that contact silibinin are shown. Catalytic residues are marked in red whereas those involved in the mechanism of action of nucleotide analogs are in green.

Table S7. Details of the interaction of remdesivir docked to SARS-CoV-2 nsp12 (6M71)

Cluster number	∆G, [kcal/mol]	Dissoc. constant, [µM]	Members	Residues of the nsp12 (6M71) that contact remdesivir
1	-7.864	1.72033	3%	LEU 172, ARG 249, VAL 315, SER 318, THR 319, PRO 323, ARG 349, GLU 350, THR 394, CYS 395, PHE 396, ARG 457, ASN 459, LEU 460, PRO 461, THR 462, ASN 628, MET 629, SER 664, PRO 677
2	-7.523	3.05892	14%	ASP 164, VAL 166, GLU 167, ARG 553, TRP 617, ASP 618, TYR 619, PRO 620, LYS 621, CYS 622, ASP 623, ARG 624, SER 759, ASP 760, ASP 761, VAL 792, PHE 793, MET 794, SER 795, LYS 798, TRP 800, GLU 811, PHE 812, CYS 813, SER 814, GLN 815
3	-7.188	5.38426	2,0%	ASP 164, VAL 166, LYS 551, TRP 617, ASP 618, TYR 619, PRO 620, LYS 621, ASP 760, ASP 761, PHE 793, SER 795, LYS 798, CYS 799, TRP 800, GLU 811, PHE 812, CYS 813, SER 814
4	-7.092	6.33133	1%	ASP 452, TYR 455, MET 542, ARG 553, ALA 554, ARG 555, THR 556, VAL 557, LYS 621, CYS 622, ASP 623, ARG 624, THR 680, SER 682, THR 687, ALA 688, ASN 691, SER 759, ASP 760

For the best-docked remdesivir molecule of each cluster, the Gibbs free energy, the dissociation constant, the number of molecule members (as %), and the residues that contact silibinin are shown. Catalytic residues are marked in red whereas those involved in the mechanism of action of nucleotide analogs are in green.

Table S8. Details of the interaction of remdesivir docked to SARS-CoV-2 nsp12 (7BTF)

Cluster number	ΔG, [kcal/mol]	Dissoc. constant, [µM]	Members	Residues of the nsp12 (7BTF) that contact remdesivir
1	-7.544	2.95240	16%	TYR 455, LYS 551, ARG 553, ARG 555, ASP 618, TYR 619, PRO 620, LYS 621, CYS 622, ASP 623, ARG 624, THR 680, SER 681, SER 682, THR 687, ALA 688, ASN 691, LEU 758, SER 759, ASP 760, LYS 798
2	-7.453	3.44254	1%	LEU 172, THR 246, ARG 249, VAL 315, SER 318, THR 319, PRO 323, ARG 349, GLU 350, THR 394, CYS 395, PHE 396, TYR 453, TYR 456, ARG 457, ASN 459, LEU 460, PRO 461, ASN 628, VAL 675, LYS 676, PRO 677
3	-7.453	3.44254	1%	VAL 588, THR 591, SER 592, LYS 593, TRP 598, MET 601, LEU 758, PHE 812, CYS 813, GLN 815, PRO 832, ARG 836, ILE 837, ALA 840, ARG 858, SER 861, LEU 862, ASP 865, MET 924, TYR 925
4	-7.172	5.53165	1%	ASP 452, TYR 455, LYS 551, ARG 553, ARG 555, ASP 618, TYR 619, PRO 620, LYS 621, CYS 622, ASP 623, ARG 624, THR 687, ASN 691, SER 759, ASP 760, ASP 761, LYS 798
5	-7.053	6.76211	1%	ASN 497, LYS 500, SER 501, LYS 545, ARG 553, ARG 555, VAL 557, ALA 558, GLY 559, VAL 560, CYS 622, ASP 623, THR 680, SER 682, GLY 683, ASP 684, ALA 685, THR 687, ALA 688, TYR 689, ASN 691, SER 759, ASP 760

For the best-docked remdesivir molecule of each cluster, the Gibbs free energy, the dissociation constant, the number of molecule members (as %), and the residues that contact silibinin are shown. Catalytic residues are marked in red whereas those involved in the mechanism of action of nucleotide analogs are in green.

Table S9. Details of the interaction of ribavirin docked to SARS-CoV-2 nsp12 (6M71)

Cluster number	ΔG, [kcal/mol]	Dissoc. constant, [µM]	Members	Residues of the nsp12 (6M71) that contact ribavirin
1	-6.262	25.69763	4%	TYR 456, MET 542, LYS 545, ARG 553, ARG 555, THR 556, VAL 557, ALA 558, ASP 623, ARG 624, LYS 676, THR 680, SER 681, SER 682, GLY 683
2	-6.241	26.62480	6%	VAL 315, SER 318, ARG 349, GLU 350, ASN 459, LEU 460, PRO 461, THR 462, ASN 628, SER 664, VAL 675, PRO 677
3	-6.216	27.77228	9%	GLY 616, TRP 617, ASP 618, TYR 619, SER 759, ASP 760, ASP 761, ALA 762, VAL 763, LYS 798, TRP 800, GLU 811, PHE 812

For the best-docked ribavirin molecule of each cluster, the Gibbs free energy, the dissociation constant, the number of molecule members (as %), and the residues that contact silibinin are shown. Catalytic residues are marked in red whereas those involved in the mechanism of action of nucleotide analogs are in green.

Table S10. Details of the interaction of ribavirin docked to SARS-CoV-2 nsp12 (7BTF)

Cluster number	∆G, [kcal/mol]	Dissoc. constant, [µM]	Members	Residues of the nsp12 (7BTF) that contact ribavirin
1	-6.194	28.82291	9%	ASP 452, ARG 553, ARG 555, THR 556, ASP 623, ARG 624, THR 680, SER 681, SER 682, THR 687, ASN 691
2	-6.149	31.09734	9%	VAL 315, ARG 349, GLU 350, ASN 459, LEU 460, PRO 461, THR 462, PRO 627, ASN 628, MET 629, PRO 677, GLY 678
3	-5.846	51.85912	1%	ILE 494, VAL 495, ASN 496, ASN 497, LYS 500, ARG 569, GLN 573, LEU 576, LYS 577, ALA 580, GLY 590, ALA 685, TYR 689
4	-5.434	103.95017	1%	VAL 588, ILE 589, GLY 590, THR 591, LYS 593, TRP 598, MET 601, LEU 758, CYS 813, GLN 815, ASP 865, MET 924, TYR 925

For the best-docked ribavirin molecule of each cluster, the Gibbs free energy, the dissociation constant, the number of molecule members (as %), and the residues that contact silibinin are shown. Catalytic residues are marked in red whereas those involved in the mechanism of action of nucleotide analogs are in green.

Table S11. Details of the interaction of sofosbuvir docked to SARS-CoV-2 nsp12 (6M71)

Cluster number	∆G, [kcal/mol]	Dissoc. constant, [µM]	Members	Residues of the nsp12 (6M71) that contact sofosbuvir
1	-7.582	2.76898	2%	ARG 249, VAL 315, SER 318, THR 319, PRO 323, ARG 349, GLU 350, PHE 396, ARG 457, ASN 459, LEU 460, PRO 461, THR 462, ASN 628, PRO 677
2	-7.195	5.32102	15%	ASP 452, TYR 455, LYS 545, ARG 553, ARG 555, THR 556, ASP 618, TYR 619, PRO 620, LYS 621, CYS 622, ASP 623, ARG 624, SER 682, ASP 760, LYS 798
3	-7.048	6.81942	1%	ILE 494, ASN 496, LYS 500, ARG 569, GLN 573, LEU 576, LYS 577, ALA 580, ILE 589, GLY 590, SER 682, GLY 683, ASP 684, ALA 685, THR 687, ALA 688, TYR 689, LEU 758, SER 759

For the best-docked sofosbuvir molecule of each cluster, the Gibbs free energy, the dissociation constant, the number of molecule members (as %), and the residues that contact silibinin are shown. Catalytic residues are marked in red whereas those involved in the mechanism of action of nucleotide analogs are in green.

Table S12. Details of the interaction of sofosbuvir docked to SARS-CoV-2 nsp12 (7BTF)

Cluster number	ΔG, [kcal/mol]	Dissoc. constant, [µM]	Members	Residues of the nsp12 (7BTF) that contact sofosbuvir
1	-7.435	3.54873	20%	ASP 452 A TYR 455 A ARG 553 A ALA 554 A ARG 555 A THR 556 A ASP 618 A TYR 619 A LYS 621 A CYS 622 A ASP 623 A ARG 624 A THR 680 A SER 682 A THR 687 A ASN 691 A ASP 760

For the best-docked sofosbuvir molecule of each cluster, the Gibbs free energy, the dissociation constant, the number of molecule members (as %), and the residues that contact silibinin are shown. Catalytic residues are marked in red whereas those involved in the mechanism of action of nucleotide analogs are in green.

Figure S1.

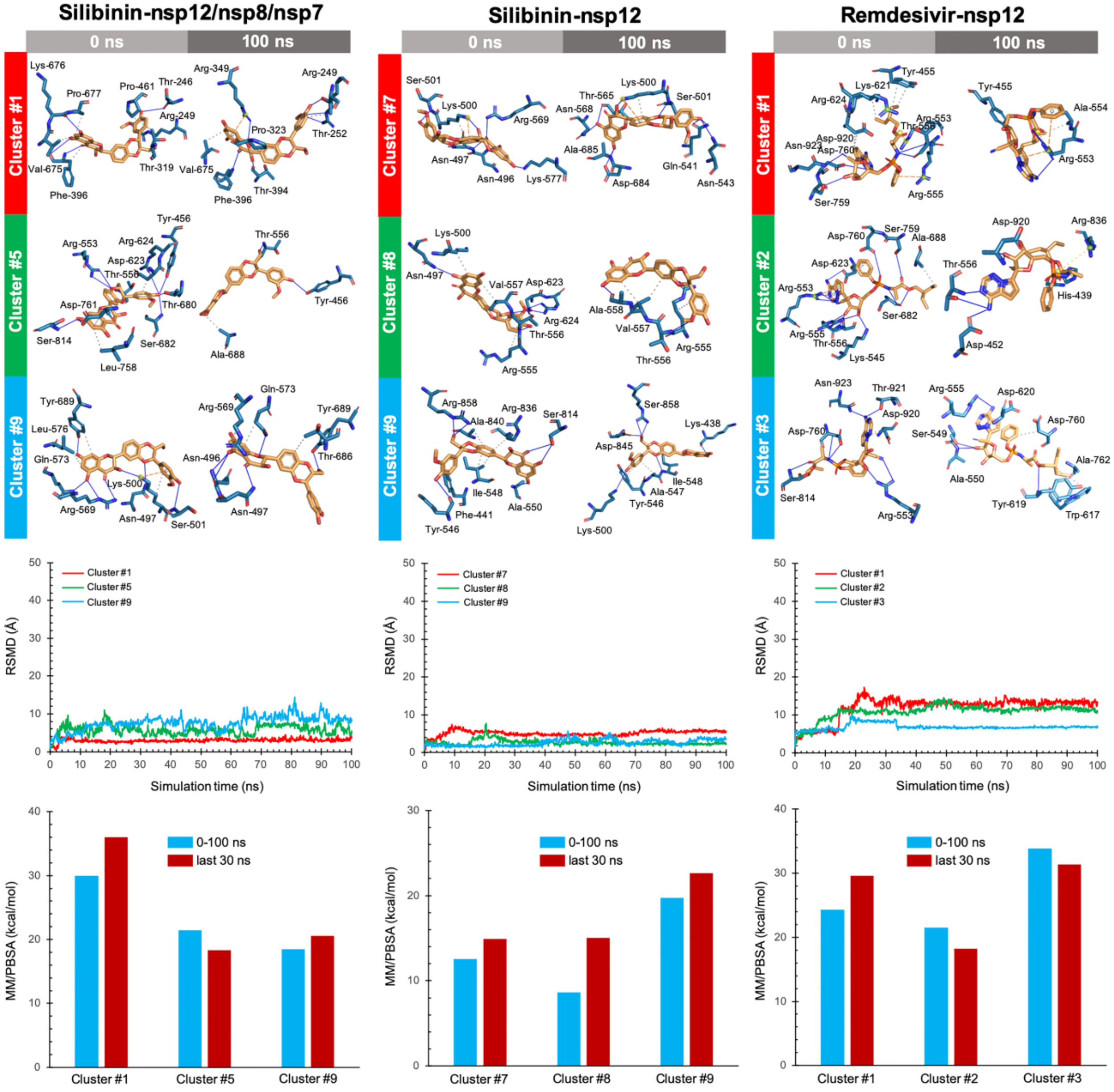
Silibinin-nsp12/nsp8/nsp7
Silibinin-nsp12
Remdesivir-nsp12
0 ns
100 ns
Cluster #1
Cluster #5
Cluster #9
Cluster #7
Cluster #8
Cluster #1
Cluster #2
Cluster #3
RSMD (Å)
Simulation time (ns)
MM/PBSA (kcal/mol)
0-100 ns
last 30 ns

Figure S1. Incorporation models of predicted RdRp-targeted silibinin and remdesivir clusters. Each inset shows the detailed interactions of silibinin and remdesivir clusters before (0 ns) and after 100 ns of molecular dynamics simulations on the 6NUR-based homology model of SARS-CoV-2 viral polymerase RdRp, indicating the participating amino acids involved in the interaction and the type of interaction (hydrogen bonds, hydrophilic interactions, salt bridges, Π-stacking, etc). Root mean square deviation (RMSD, Å) of silibinin/remdesivir heavy atoms over simulation time, measured after superposing the homology model of SARS-CoV-2 RdRp protein on its reference structure is shown. Molecular Mechanics/Poisson-Boltzmann Surface Area (MM/PBSA) free energy analysis of the SARS-CoV-2 RdRp protein forming a complex with silibinin/remdesivir using YASARA dynamics v19.9.17 software. The best-docked complex as the initial conformation for MD simulation followed by 1000 snapshots (100 ns) obtained from the MD trajectory were employed to calculate the values of free energy binding of each luster. Additionally, the average value calcu lated for the last 300 snapshots (30 ns) is also displayed. YASARA-calculated binding energy provides positive values when the predicted binding is strong and stable whereas negative values indicate no binding. Figures were prepared using PyMol 2.3 software.
